# Fish oil supplementation of rats fed a high fat diet during pregnancy improves offspring insulin sensitivity

**DOI:** 10.3389/fnut.2022.968443

**Published:** 2022-09-02

**Authors:** Vidit V. Satokar, Mark H. Vickers, Clare M. Reynolds, Anna P. Ponnampalam, Elwyn C. Firth, Manohar L. Garg, Carolyn J. Barrett, Wayne S. Cutfield, Benjamin B. Albert

**Affiliations:** ^1^Liggins Institute, University of Auckland, Auckland, New Zealand; ^2^Conway Institute of Biomolecular and Biomedical Research, University College Dublin, Dublin, Ireland; ^3^Manaaki Mānawa – The Centre for Heart Research, Department of Physiology, University of Auckland, Auckland, New Zealand; ^4^Nutraceuticals Research Program, School of Biomedical Sciences and Pharmacy, University of Newcastle, Callaghan, NSW, Australia; ^5^A Better Start – National Science Challenge, University of Auckland, Auckland, New Zealand

**Keywords:** fish oil, omega-3, rat, pregnancy, developmental programming, DOHaD, metabolism, insulin sensitivity

## Abstract

**Introduction:**

In rats, a maternal high-fat diet (HFD) leads to adverse metabolic changes in the adult offspring, similar to the children of mothers with obesity during pregnancy. Supplementation with a high dose of fish oil (FO) to pregnant rats fed a HFD has been shown to prevent the development of insulin resistance in adult offspring. However, the effects of supplementation at a translationally relevant dose remain unknown.

**Aim:**

To determine whether supplementation with a human-relevant dose of FO to pregnant rats can prevent the long-term adverse metabolic and cardiovascular effects of a maternal HFD on adult offspring.

**Methods:**

Female rats (*N* = 100, 90 days of age) were assigned to HFD (45% kcal from fat) or control diet (CD) for 14 days prior to mating and throughout pregnancy and lactation. Following mating, dams received a gel containing 0.05 ml of FO (human equivalent 2–3 ml) or a control gel on each day of pregnancy. This produced 4 groups, CD with control gel, CD with FO gel, HFD with control gel and HFD with FO gel. Plasma and tissue samples were collected at day 20 of pregnancy and postnatal day 2, 21, and 100. Adult offspring were assessed for insulin sensitivity, blood pressure, DXA scan, and 2D echocardiography.

**Results:**

There was an interaction between maternal diet and FO supplementation on insulin sensitivity (*p* = 0.005) and cardiac function (*p* < 0.01). A maternal HFD resulted in impaired insulin sensitivity in the adult offspring (*p* = 0.005 males, *p* = 0.001 females). FO supplementation in the context of a maternal HFD prevented the reduction in insulin sensitivity in offspring (*p* = 0.05 males, *p* = 0.0001 females). However, in dams consuming CD, FO supplementation led to impaired insulin sensitivity (*p* = 0.02 males, *p* = 0.001 females), greater body weight and reduced cardiac ejection fraction.

**Conclusion:**

The effects of a human-relevant dose of maternal FO on offspring outcomes were dependent on the maternal diet, so that FO was beneficial to the offspring if the mother consumed a HFD, but deleterious if the mother consumed a control diet. This study suggests that supplementation with FO should be targeted to women expected to have abnormalities of metabolism such as those with overweight and obesity.

## Introduction

The prevalence of obesity has increased dramatically worldwide (1) with approximately 60% of women of reproductive age now overweight or obese (2, 3). While this has direct impacts on the health of women, it also has important implications for future generations of children. Maternal pre-pregnancy obesity is one of the strongest predictors for obesity in childhood (4) and later life, and is associated with unfavorable changes in metabolism including greater insulin resistance (5–7), triglyceride concentrations (6, 7), and blood pressure (7). Thus, maternal obesity can perpetuate transmission of obesity and related metabolic disorders across generations (8) and there remains an urgent need for intervention strategies to break this cycle of disease.

Women with obesity are more likely to have systemic and adipose tissue inflammation (9) and, during pregnancy, this leads to exaggeration of insulin resistance in the second half of pregnancy (9) and excess delivery of glucose and fatty acids to the fetus (5). This is thought to lead to greater metabolic risk through lifelong alterations in gene expression (10, 11) mediated by epigenetic changes (12–15). As the n-3 PUFAs found in fish oil (FO) are anti-inflammatory and insulin-sensitizing (16–19), supplementation of women with overweight or obesity may reduce the risk of obesity and metabolic disorders in the offspring.

In rodent models, a maternal high fat diet (HFD) in pregnancy represents a model with similar phenotypic outcomes to that of human pregnancy complicated by obesity. Pregnant rats fed a HFD develop greater adiposity and insulin resistance (20), and the offspring develop an adverse phenotype of hyperphagia (21), greater adiposity and body weight (20, 21), insulin resistance (20–22), high blood pressure (21), and dyslipidemia (21, 22). Effects of maternal HFD on offspring cardiac morphology and function have also been described. A maternal HFD is associated with greater heart size (23–25), cardiac lipid droplet accumulation (23, 24) and lipid peroxidation (23) in neonatal rats. These adverse changes in cardiac morphology appear to be mediated by insulin resistance with hyperinsulinaemia in the offspring leading to mitogenic effects on the heart (26). Limited evidence in mice shows that greater heart size (26, 27) and lipid accumulation persists to adulthood (27), but long term effects on cardiac function have not been assessed.

Fish oil supplementation increases insulin sensitivity (28) and lowers triglycerides in rats (29). We have previously shown that supplementing a high dose of FO by oro-gastric gavage to rats fed a HFD during pregnancy prevented the development of impaired insulin sensitivity in adult male offspring (17). However, many aspects of the adverse metabolic phenotype were not prevented and the dose of fish oil was too great to translate to human pregnancy. We speculated that if the fish oil had been continued through lactation and delivered using a less invasive methodology there would be greater metabolic benefits, to offspring of both sexes. Therefore, we conducted a trial of FO supplementation in the context of maternal HFD during pregnancy and lactation, using oil-emulsified gels to administer a lower (human-relevant) dose of FO.

## Methods

### Animal ethics

Ethical approval was granted by the Animal Ethics Committee at the University of Auckland (approval #R001936). The study was performed in accordance with all appropriate institutional and international guidelines and regulations for animal research and is reported according to the ARRIVE guidelines.

### Study design

Female Sprague-Dawley rats (*n* = 100) were obtained at weaning (21 days) and housed under standard conditions at 25°C with a 12-h light:12-h dark cycle and fed a standard chow diet (Harlan-Teklad Diet 2018, Oxon, United Kingdom) *ad libitum*. Pre-mating body weights were monitored twice weekly.

**FIGURE 1:**
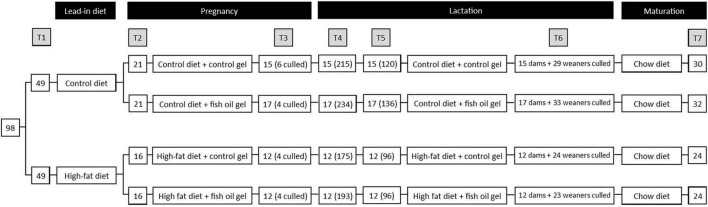
Study design. T1: Initial randomization to diet, T2: Mating and randomization to gel treatment, T3: subset of dams culled on day 20 of pregnancy, continued dam number (culled dam number), T4: Pups delivered, dam number (pup number), T5: Litters standardized on postnatal day 2, dam number (pup number), T6: dams and weaners culled on postnatal day 21. 1 male and 1 female weaner per litter utilized for adult assessments. T7: adult male and female offspring culled at day 100, number represents total of male and female pups. Note that 1 pair of cohoused female offspring in the Con-FO group were excluded due to misattribution of sex that led to pregnancy.

A model of maternal obesity was utilized as previously reported by our group in which a maternal obesogenic diet leads to obesity in offspring, independent of postnatal diet (20). At day 90, rats were assigned to one of two isocaloric diets *ad libitum* for 14 days prior to mating (Figure 1): a HFD (D12451, Research Diets Inc., New Brunswick, NJ, United States) containing 45% kcal as fat, or a matched control diet (Con, D12450H) containing 10% kcal as fat (Table 1). Neither diet contained the long chain n-3 PUFAs (eicosapentaenoic acid (EPA) or docosahexaenoic acid (DHA)). At least 5 days prior to mating a single 5 ml control gel, which was raspberry flavored but contained no FO, was placed into their food hopper in the morning to acclimatize animals.

**TABLE 1:** Nutritional content of control and high-fat diets provided to rat dams during pregnancy and lactation.

Macronutrient	Description	Unit	Control diet (D12450H)	High-fat diet (D12451)
Carbohydrate	Sucrose	% kcal	17	17
	Other carbohydrates	% kcal	53	18
Protein	Total	% kcal	20	20
Fat	Total	% kcal	10	45
	Saturated fats	% of total fat (wt)	22.7	31.4
	Monosaturated fats	% of total fat (wt)	29.9	35.5
	Polyunsaturated fats	% of total fat (wt)	47.4	33.1
	Eicosapentaenoic acid (EPA)	% of total fat (wt)	0	0
	Docosahexaenoic acid (DHA)	% of total fat (wt)	0	0
Total energy		kcal/gm	3.85	4.73

Diets were sourced from Research Diets, Inc.

Rats were time-mated using an estrous cycle monitor (EC-40, Fine Science Tools, San Francisco, CA, United States). *Day 1* of pregnancy was determined by detection of spermatozoa following vaginal lavage, and thereafter pregnant rats were individually housed. Immediately following successful mating, pregnant rats in both the control diet and HFD groups were further allocated to one of the two treatment gels in a 2 × 2 balanced design. The treatment gels were raspberry flavored and contained either 50 μl of FO or no FO (control). Thus, the study had four treatment groups: Con-Con, in which dams consumed a control diet and control gel, Con-FO, control diet and a gel with 50 μl of FO, HF-Con, HFD and control gel, HF-FO, in which dams consumed HFD and a gel with 50 μl of FO. 50 μl of FO in pregnant dams is equivalent to 2–3 ml taken by a woman, adjusted for body surface area (30). Each morning of pregnancy a single gel (appropriate for the study group) was placed in one side of the food hopper, with the food shifted to the other side. Rats received the gel treatment on each day of pregnancy and for the 21 days of lactation. Maternal food intake and body weight were recorded every 3rd day throughout pregnancy and lactation.

### Fish oil supplement

The FO supplement consisted of refined FOs produced from anchovy, sardines, mackerel, herring, and tuna (New Zealand Health Manufacturing, Auckland, New Zealand). Independent verification of n-3 PUFA content and oxidative state (Table 2) was undertaken using the methodology previously described (31). The oil had a peroxide value of 5.2 meq/kg and anisidine value of 4.2 meq/kg. This peroxide value indicates a relatively low level of oxidation, equivalent to the upper limit of the Global Organization for EPA and DHA industry guidelines, and within the recommendations of the Australian Therapeutic Goods Administration and European pharmacopeia (32). The FO was separated into 15ml aliquots, frozen at −20°C, sealed, and stored in darkness. Individual aliquots were thawed to room temperature prior to producing the gels.

**TABLE 2:** Fatty acid composition and oxidative quality of the fish oil supplement.

	Fish oil
**Fatty acid concentration, mg/g of oil**	
C14:0 (Myristic acid)	86.2 (0.9)
C16:0 (palmitic acid)	176.8 (0.2)
C16:1 (palmitoleic acid)	94.4 (0.7)
C18:0 (stearic acid)	33.2 (0.8)
C18:1n-9 (oleic acid)	55.8 (0.4)
C18:1n-7 (cis-11-octadecenoic acid)	25.5 (0.1)
C18:2n-6 (linoleic acid)	7.9 (0.2)
C18:3n-6 (α—linolenic acid)	2.4 (0.2)
C18:3n-3 (γ—linolenic acid)	11.8 (0.6)
C20:0 (arachidic acid)	0
C20:1n-9 (eicosenoic acid)	9.6 (0.2)
C20:2n-6 (eicosadienoic acid)	2.1 (0.2)
C20:3n-6 (dihomo-γ-linolenic acid)	2.1 (0.1)
C20:4n-6 (arachidonic acid)	16.6 (0.5)
C20:5n-3 (eicosapentaenoic acid)	176.7 (1.9)
C22:5n-3 (docosapentaenoic acid)	16.4 (0.9)
C24:0 (tetracosanoic acid)	1.3 (0.2)
C22:6n-3 (docosahexaenoic acid)	122.1 (2.3)
C24:1 (Nervonic acid)	4.9 (0.3)
**Oxidative indices**	
Peroxide value, meq/kg	5.24 (0.01)
Anisidine value	4.2 (0.1)

Fatty acid concentrations in the fish oil supplement (mg/g of oil), determined by gas chromatography. Data are Means (SE), from analysis of 6 replicates. Oxidative indices determined from analysis of 3 replicates.

### Gel preparation

The gels were prepared according to a published protocol (33). In short, an emulsion of FO was created using a 4% non-polar starch solution (Ingredion NZ, Auckland, New Zealand), which was added to a solution of gelatin and raspberry jelly crystals and allowed to set 5 ml gels. Control gels had no oil added, while each FO gel contained 0.05 ml of the FO. Gels were stored in the refrigerator at 4°C prior to use, for a maximum of 3 days.

We have recently reported that the oil emulsified gel treatments are rapidly and completely consumed after placement in the cage and appear to be an enriching experience for the rats (33).

### Outcome measures

#### Gestational day 20 (GD20)

At day 20 of pregnancy, i.e., 1 day prior to delivery, 4–6 dams from each group were fasted overnight and anesthetized by isoflurane, followed by decapitation (T2, Figure 1). The uterine horns were opened, and fetuses counted and sexed (using anogenital distance). Maternal liver and retroperitoneal fat depots were collected. Body weight and placental weight were recorded from one male and one female fetus. Fetuses were examined, beginning in the central uterus, and the first male and first female identified were weighed and the liver and placenta were collected. In addition, trunk blood was collected from both dams and fetuses in heparinized tubes and stored on ice until separation of plasma for analysis. Fasting blood glucose concentrations were determined from tail blood from the mothers and *via* trunk blood from fetuses using a glucometer (Accu-Chek Performa, Roche).

#### Birth and postnatal day 2 (PN2)

At birth, offspring were counted, sexed, weighed, and length measured (NA; nose to anus). On postnatal day 2, offspring were weighed again and randomly culled to produce litters of 8 (targeting 4 males and 4 females where possible) to standardize nutrition until weaning. Pups not allocated to litters were killed by decapitation, and plasma, red blood cells, and liver were collected for analysis. Dam and pup weights, and dam food consumption were recorded every 3rd day from PN2 until weaning.

#### Weaning

At postnatal day 21 (PN21), following an overnight fast, dams were culled using sodium pentobarbitone anesthesia (60 mg/kg intraperitoneal) followed by decapitation. Blood was collected in heparinized tubes and centrifuged for collection of plasma and red blood cells for analysis. Liver and the gonadal and retroperitoneal fat depots were collected and weighed. The offspring were culled to retain 1 male and 1 female offspring per dam. They were housed in sex-matched pairs (either an animal of the same sex, from the same group, or a non-studied cage-mate) and fed a standard chow diet (Harlan-Teklad Diet 2018, Oxon, United Kingdom) *ad libitum*. Body weight and food consumption were recorded every 3rd day until the final assessment in adulthood (day 100).

#### Adult assessments

At day 70, offspring were fasted overnight. The following morning their food was returned, and their post-fast food consumption was determined by weighing the food left in the hopper 24 h later. At day 80, systolic blood pressure of the male adult offspring was measured by tail cuff plethysmography (Model 179 with an automatic cuff inflation pump (NW20), IITC, Life Science, Woodland Hills, CA, United States), according to the manufacturer’s instructions (34). Prior to blood pressure recordings, the animals were warmed and acclimatized to the restraint tube for 15 min. Mean blood pressure was calculated by taking three clear pressure recordings per animal (coefficients of variation (CV) of repeated measurements <5%). Blood pressure could not be reliably measured in female offspring, as appropriately sized equipment was not available.

At day 90, body composition was quantified using a dual x-ray absorptiometry scan (Lunar iDXA, GE Medical Systems, Chicago, IL, United States), while animals were under light isoflurane anesthesia. Following the DXA scan, transthoracic echocardiography was performed on a subgroup of offspring (*n* = 6–8) using a Vevo 3100 high-resolution imaging system (Fujifilm VisualSonics, Inc.), paired with a 21-MHz transducer (MS250). Imaging data was captured for analysis using VevoStrain software. B-mode cine loops were used in parasternal long- and short-axis view to assess basic variables for systolic function. Imaging data was peer-reviewed for quality regarding differentiation of wall borders and general absence of artifacts. M-mode was used to measure cardiac wall and chamber dimensions. Parameters including left ventricular structure, systolic and diastolic chamber diameters, fractional shortening, ejection fraction, cardiac output were assessed and measured.

At day 95, glucose tolerance was assessed using an oral glucose tolerance test (OGTT) following a 6-h fast. Prior to giving a glucose load (2 g/kg of body weight), tail blood was collected (T0). Further blood was collected from the tail at T15, T30, T60, T90, and T120. Samples were collected and stored for the measurement of plasma insulin. The insulin and glucose concentrations at T0, and T30–120 were used to derive the Matsuda Index, which was the primary outcome and is a highly repeatable measure (35) and closely correlated with the hyperinsulinaemic euglycemic clamp (*r* = 0.77) in humans (36) and its use has been reported in rats (37, 38).


(1) $$\mathrm{Matsuda}\,\mathrm{Index}=\frac{10000}{\sqrt{\begin{array}{c} \left(\mathrm{fasting}\mathrm{glucose}*\mathrm{fasting}\mathrm{insulin}\right)(\mathrm{mean}\mathrm{glucose}*\mathrm{mean}\\ \mathrm{insulin}) \end{array}}}$$

In addition, HOMA-IR, which is validated and widely used in rats (39), was calculated as well as the glucose and insulin area under the curve (AUC) using the trapezoidal method.


(2) $$\text{HOMA}-\text{IR}=\frac{\mathrm{fasting}\,\mathrm{insulin}*\mathrm{fasting}\,\mathrm{glucose}}{22.5}$$

At day 100 (PN100), adult male and female offspring were fasted overnight and culled as details above for mothers at PN21. Liver, heart, and retroperitoneal fat were weighed, while liver, heart, gonadal fat and plasma (separated from heparinized trunk blood) were collected for analysis.

### Biochemical analyses

Glucose concentrations were measured from the tail blood sample at the time of the OGTT using a glucometer (Roche Accuchek Performa ASPAC MMOLmeter). Insulin, leptin, and adiponectin were measured by rat-specific ELISA (Crystal Chem, IL, United States) with CV’s of 9, 7, and 4%, respectively. Alkaline phosphatase (ALP), alanine transaminase (ALT), aspartate transaminase (AST), free fatty acids, triglycerides, high-density lipoprotein cholesterol (HDL-C), low-density lipoprotein cholesterol (LDL-C), and total cholesterol were measured on Hitachi 902 autoanalyzer (Hitachi High Technologies, Tokyo, Japan) with CV’s less than 6%.

### Primary outcome and power analysis

The Primary Outcome was the Matsuda index of insulin sensitivity in the adult offspring. Given previous data showing a maternal HFD was associated with mean fasting insulin of 4.89 ng/ml and standard deviation of 0.926 in the adult male offspring (20), a sample of approximately 12 animals per group was required to have 90% power to detect a 25% change in fasting insulin (17). Two major comparisons were required: (1) HF-Con vs. Con-Con to demonstrate the specific effect of the maternal HFD during pregnancy and lactation on insulin sensitivity in the adult offspring; (2) HF-FO and HF-Con to investigate the specific effect of the FO supplement, in the context of a maternal HFD.

### Statistical analysis

All outcomes were tested for normality (Shapiro-Wilk). Insulin and triglyceride concentrations were log-normalized prior to analysis. All outcomes were compared between groups using two-way ANOVA and *post hoc* comparisons using the Holm-Sidak’s correction, with maternal diet and FO supplementation as factors. The interaction between diet and treatment was examined in all models. All statistical analyses were carried out using SigmaStat software (Systat software, San Jose, CA, United States).

GraphPad Prism 8 (GraphPad Software, United States) was used to generate figures. Significance was maintained at *p* < 0.05 (2-tailed test significance). Data are represented as means ± SEM unless otherwise stated.

## Results

Of the 100 dams, 2 were excluded prior to mating due to excessive weight loss (weight > 2SD below the mean), 49 were allocated to control diet and 49 to the HFD. All dams in the control diet group and 40 from the HFD group had mating confirmed and allocated to treatment groups. However, 7 from the control diet group (3 from Con-Con and 4 from Con-FO) and 8 from HFD group (5 from HF-Con and 3 from HF-FO) were subsequently found to not be pregnant. This resulted in 74 successful mating comprising 21 dams in Con-Con, 21 in Con-FO, 16 in HF-Con, and 16 in HF-FO (Figure 1).

**FIGURE 2:**
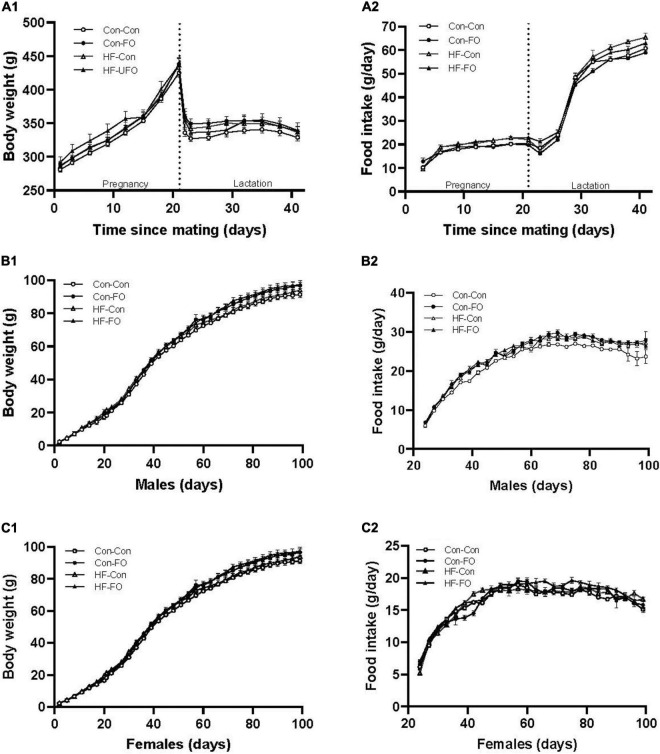
Weight, food consumption, and energy intake in rat dams **(A)**, male **(B)**, and female offspring **(C)** according to the maternal diet (control or high-fat diet) and gel treatment during pregnancy [fish oil or control (no oil)]. **(A)** body weight **(A1)**, food consumption **(A2)** of dams from mating (day 0) through to weaning (day 42). **(B)** body weight **(B1)**, food consumption **(B2)** of male offspring from weaning through to adulthood (day 100). **(C)** body weight **(C1)**, food consumption **(C2)** of female offspring from weaning through to adulthood (day 100). Error bars represent standard errors.

During pregnancy, all gel treatments were completely consumed. Neither diet nor the gel treatment affected the number of offspring per litter or the sex ratio (Table 3). Weight gain and food consumption during pregnancy did not differ between groups (Figure 2A). However, although there was no difference in the maternal weights at the end of lactation, the caloric intake was higher in the HF-Con group (0.62 vs. 0.53 kcal/g/day, *p* = 0.0001) than the Con-Con group. Further, there was a significant effect of diet on adiposity of dams, so that across the high fat diet groups, there was greater retroperitoneal fat at PN21 (*p* = 0.03) and a trend to greater fat during pregnancy (GD20, *p* = 0.08) (Tables 4, 5).

**TABLE 3:** Maternal weight and metabolic parameters at key milestones of the study and litter characteristics at birth, according to the maternal diet (control or high-fat diet) and the gel treatment during pregnancy [fish oil or control (no oil gel)].

	Maternal control diet		Maternal high-fat diet		Effect of diet	Effect of treatment	Diet vs. treatment interaction
			
	Control	Fish oil	Control	Fish oil	p^1^	p^1^	p^1^
*n*	21	21	16	16			
**Maternal weight (g)**							
Prior to diet allocation (day −14)	269.3 (3.8)	270.8 (5.5)	260.4 (6.0)	266.3 (8.0)	0.75	0.52	0.69
Mating (day 1)	280.9 (3.7)	283.9 (4.3)	291.5 (7.9)	284.4 (6.3)	0.84	0.83	0.87
End of pregnancy (day 18)	386.6 (5.0)	389.0 (7.5)	390.1 (8.1)	400.6 (11.9)	0.54	0.57	0.78
End of lactation (day 42)	328.8 (5.5)	336.4 (6.9)	337.5 (8.5)	331.8 (8.5)	0.54	0.56	0.63
Pregnancy weight gain %	28.9 (0.6)	30.0 (1.1)	32.9 (1.2)	31.3 (1.5)	0.37	0.22	0.42
**Maternal food intake**							
Pregnancy food intake (kcal/g/day)	0.23 (0.01)	0.23 (0.01)	0.25 (0.01)	0.25 (0.01)	**0.002**	0.93	0.76
Postnatal food intake (kcal/g/day)	0.52 (0.01)	0.48 (0.01)	0.62 (0.02)**	0.61 (0.02)	**0.001**	0.33	0.09
**Birth characteristics**							
Litter size (n)	16.4 (1.2)	14 (0.6)	14.7 (0.5)	14.9 (0.4)	0.69	0.15	0.58
Sex ratio (% male per litter)	49.8 (0.2)	44.4 (0.2)	52 (0.4)	57.0 (0.1)	0.71	0.45	0.46
Weight of male pups (g)	6.3 (0.1)	6.6 (0.1)*	6.4 (0.1)	6.2 (0.1)	**0.003**	0.26	**0.001**
Length of male pups (mm)	48.9 (0.2)	49.7 (0.3)	49.1 (0.3)	48.9 (0.2)	0.14	0.22	0.08
Weight of female pups (g)	6.1 (0.1)	6.3 (0.1)	6.0 (0.1)	5.8 (0.1)	**0.001**	0.70	**0.03**
Length of female pups (mm)	48.3 (0.2)	48.4 (0.3)	47.6 (0.3)	47.1 (0.6)	**0.001**	0.81	0.83
**Day 2 culls**							
**Male pups**	21	20	16	16			
Weight (g)	6.8 (0.2)	7.0 (0.2)	6.9 (0.3)	6.9 (0.3)	0.83	0.63	0.98
Random blood glucose (mmol/l)	5.1 (0.2)	5.5 (0.3)	4.9 (0.2)	5.1 (0.3)	0.19	0.40	0.70
Liver weight (% body weight)	3.6 (0.2)	3.7 (0.1)	4.0 (0.1)	3.8 (0.2)	0.96	0.10	0.35
**Female pups**							
Weight (g)	6.5 (0.2)	6.8 (0.3)	6.6 (0.2)	6.6 (0.3)	0.50	0.59	0.63
Random blood glucose (mmol/l)	5.1 (0.1)	5.1 (0.3)	5.1 (0.2)	5.2 (0.3)	0.63	0.72	0.66
Liver weight (% body weight)	3.8 (0.1)	4.0 (0.1)	5.3 (0.4)*	3.7 (0.2)^††^	0.31	0.59	0.36

Weight, blood glucose and liver weight percentage of the total body weight of the male and female pups culled on postnatal day 2. Data are Means (SE). p values ≤ 0.05 are bold. *p < 0.05; **p < 0.005 vs. Con-Con, ^††^*p* < 0.005 vs. HF-Con—Post hoc tests (Holm-Sidak’s correction). p^1^ represents *p*-value of two-way ANOVA with factors including diet and treatment interaction.

**TABLE 4:** Characteristics and metabolic parameters of dams culled on day 20 of pregnancy.

	Maternal control diet		Maternal high-fat diet		Effect of diet	Effect of treatment	Diet vs. treatment interaction
			
	Control	Fish oil	Control	Fish oil	p^1^	p^1^	p^1^
*n*	6	4	4	4			
**Maternal characteristics**							
Weight	445.2 (9.4)	433.5 (16.4)	437.0 (17.3)	479.1 (28.5)	0.31	0.41	0.15
Liver weight (% bw)	3.7 (0.1)	3.6 (0.1)	3.4 (0.2)	3.3 (0.2)	**0.03**	0.65	0.96
Retroperitoneal fat (% bw)	1.2 (0.2)	1.3 (0.2)	1.6 (0.2)	1.5 (0.1)	0.08	0.76	0.79
**Glucose homeostasis**							
Fasting glucose (mmol/l)	6.3 (0.3)	6.5 (0.5)	6.5 (0.4)	6.4 (0.1)	0.64	0.07	0.91
Fasting insulin (ρmol/l)	257 (32)	392 (26)*	291 (12)	285 (16)	**0.04**	**0.004**	**0.003**
HOMA-IR	4.0 (0.6)	6.3 (0.5)*	4.6 (0.3)	4.1 (0.3)	**0.05**	**0.04**	**0.006**
**Other**							
CRP (mg/l)	0.03 (0.01)	0.04 (0.01)	0.04 (0.02)	0.02 (0.01)	0.86	0.24	0.86
Triglycerides (mmol/l)	2.2 (0.1)	3.8 (0.7)	3.3 (0.9)	2.5 (0.6)	0.86	0.50	0.07
**Male fetus**							
Weight (g)	4.1 (0.2)	4.2 (0.2)	4.2 (0.2)	4.4 (0.2)	0.49	0.45	0.86
Blood glucose (mmol/l)	2.5 (0.3)	2.6 (0.3)	2.3 (0.3)	2.3 (0.5)	0.43	0.85	0.91
Placenta weight (% bw)	10.8 (0.4)	12.1 (0.5)	12.1 (0.7)	12.0 (1.5)	0.47	0.41	0.40
Liver weight (% bw)	6.4 (0.5)	6.5 (0.5)	6.8 (0.3)	6.5 (0.4)	0.62	0.86	0.61
**Female fetus**							
Weight (g)	4.1 (0.1)	3.9 (0.1)	3.8 (0.3)	4.2 (0.1)	0.60	0.52	0.13
Blood glucose (mmol/l)	3.2 (0.5)	2.4 (0.1)	2.1 (0.3)	1.87 (0.2)	0.06	0.19	0.44
Placenta weight (% bw)	11.2 (0.7)	13.5 (1.0)	12.0 (0.5)	10.7 (0.7)	0.21	0.51	**0.03**
Liver weight (% bw)	7.4 (0.5)	7.4 (0.5)	7.1 (0.5)	6.9 (0.5)	0.53	0.82	0.90

Weight, glucose, and liver weight percentage of body weight of the male and female fetus sampled. Data are Means (SE). *P-values* ≤ 0.05 are bold. *p < 0.05 vs. Con-Con; bw, body weight. p^1^ represents *p*-value of two-way ANOVA with factors including diet and treatment interaction.

**TABLE 5:** Characteristics of mothers and weaners culled on postnatal day 21 according to the maternal diet (control or high-fat diet) and the fish supplementation during pregnancy [fish oil or control (no oil gel)].

	Maternal control diet		Maternal high-fat diet		Effect of diet	Effect of treatment	Diet vs. treatment interaction
			
	Control	Fish oil	Control	Fish oil	p^1^	p^1^	p^1^
*n*	15	17	12	12			
**Maternal characteristics**							
Weight (g)	316.1 (5.3)	325.3 (5.7)	314.5 (7.3)	326.9 (9.9)	0.99	0.13	0.82
Liver weight (% body weight)	6.4 (0.3)	5.9 (0.3)	4.1 (0.1)**	4.0 (0.1)	**0.001**	0.32	0.54
Gonadal fat (% body weight)	1.3 (0.1)	1.4 (0.1)	1.7 (0.1)	1.4 (0.2)	0.23	0.45	0.17
Retroperitoneal fat (% body weight)	0.8 (0.1)	0.8 (0.1)	1.1 (0.1)	0.9 (0.1)	**0.03**	0.19	0.60
**Glucose homeostasis**							
Fasting glucose (mmol/l)	5.7 (0.2)	5.3 (0.1)	6.3 (0.3)	6.3 (0.3)	**0.002**	0.40	0.42
Fasting insulin (pmol/l)	466 (79)	477 (77)	393 (53)	340 (51)	0.08	0.07	0.12
HOMA-IR	6.6 (1.2)	6.4 (1.1)	6.1 (0.7)	5.3 (0.8)	0.93	0.44	0.53
**Male weaners**							
Weight	56.2 (1.2)	59.7 (0.9)	65.5 (1.6)**	64.4 (1.7)	**0.001**	0.38	0.10
Liver weight (% of body weight)	3.7 (0.15)	3.7 (0.12)	3.6 (0.09)	3.6 (0.06)	0.92	0.58	0.50
Gonadal fat (% of body weight)	0.2 (0.02)	0.3 (0.01)	0.4 (0.1)**	0.4 (0.04)	**0.001**	0.67	0.42
Retroperitoneal fat (% of body weight)	0.4 (0.02)	0.4 (0.12)	0.6 (0.04)**	0.6 (0.03)	**0.001**	0.38	0.13
**Female weaners**							
Weight (g)	54.4 (0.9)	57.6 (0.9)	63.1 (1.4)**	62.3 (1.4)	**0.001**	0.32	0.08
Liver weight (% of body weight)	3.8 (0.1)	3.7 (0.1)	3.9 (0.1)	3.6 (0.1)	0.25	**0.05**	0.22
Gonadal fat (% of body weight)	0.3 (0.02)	0.3 (0.02)	0.5 (0.05)*	0.4 (0.05)	**0.001**	0.67	0.58
Retroperitoneal fat (% of body weight)	0.3 (0.02)	0.3 (0.02)	0.4 (0.03)**	0.4 (0.02)	**0.001**	0.33	0.45

Data are means (SE). *P-values* ≤ 0.05 are bold. *p < 0.05; **p < 0.005 vs. Con-Con. p^1^ represents *p*-value of two-way ANOVA with factors including diet and treatment interaction.

### Primary outcome

**FIGURE 3:**
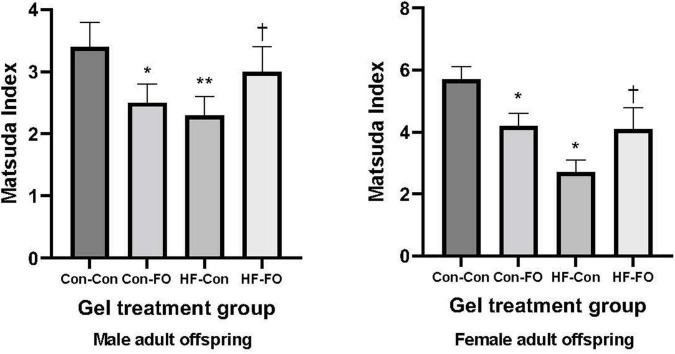
Insulin sensitivity derived by Matsuda Index in the male and female adult offspring (day 95) presented according to the maternal diet (control or high-fat diet) and the gel treatment during pregnancy [Fish oil or control (no oil gel)]. Data are Means (SE). *p < 0.05; **p < 0.005 vs. con-con ^†^*p* < 0.05 vs. HF-Con.

**FIGURE 4:**
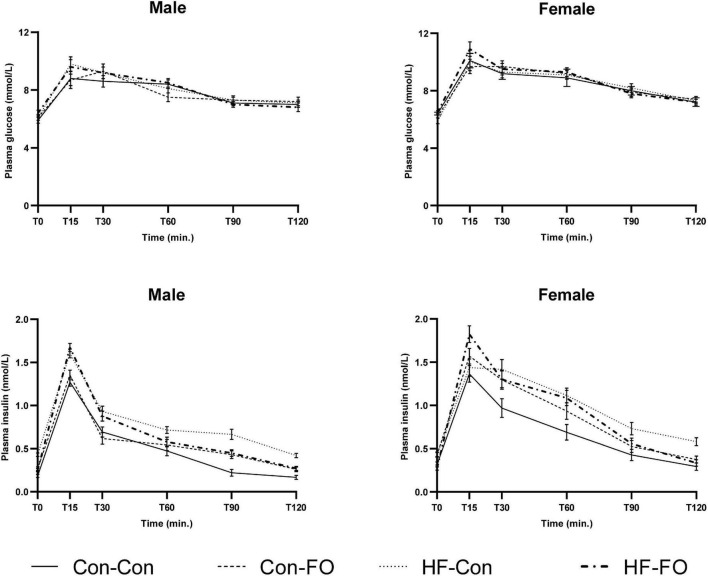
Plasma glucose (above) and insulin (below) concentrations at each time point of the oral glucose tolerance test in male and female adult offspring (day 95). Error bars represent standard errors.

There was an interaction between diet and treatment for the primary outcome (Matsuda index in adult offspring) for male (*p* = 0.0001) and female (*p* = 0.005) offspring, such that the effect of the FO intervention on insulin sensitivity of the offspring was opposite when dams consumed a control diet, when compared with the offspring of those consuming a HFD. In the adult offspring, a maternal HFD, led to lower insulin sensitivity (Matsuda index) (HF-Con vs. Con-Con; 2.3 vs. 3.4, *p* = 0.005 in males and 2.7 vs. 5.7, *p* = 0.001 in females). Maternal FO supplementation in the context of a maternal HFD led to greater insulin sensitivity in the adult offspring (HF-FO vs. HF-Con; 3.0 vs. 2.3, *p* = 0.05 in males and 4.0 vs. 2.7, *p* = 0.0001 in females). However, in the context of a maternal control diet, FO supplementation led to lower insulin sensitivity (Con-FO vs. Con-Con; 2.5 vs. 3.4, *p* = 0.02 in males and 4.2 vs. 5.7, *p* = 0.001 in females) (Figure 3 and Tables 6, 7). Glucose and insulin concentrations during the glucose tolerance test are presented in Figure 4.

**TABLE 6:** Indices of glucose metabolism obtained during oral glucose tolerance test performed on day 95 in the male adult offspring.

	Maternal control diet		Maternal high-fat diet		Effect of diet	Effect of treatment	Diet vs. treatment interaction
			
Male adult offspring	Control	Fish oil	Control	Fish oil	p^1^	p^1^	p^1^
	15	17	12	12			
Matsuda index	5.7 (0.7)	4.2 (0.5)*	2.7 (0.4)*	4.0 (1.2)^†^	**0.02**	0.10	**0.0001**
Glucose (mmol/l)							
T0	6.4 (0.1)	6.2 (0.1)	5.9 (0.2)*	6.5 (0.1)^†^	0.28	0.21	**0.01**
T15	10.1 (0.5)	9.7 (0.5)	10.0 (0.6)	10.9 (0.5)	0.34	0.73	0.21
T30	9.2 (0.4)	9.7 (0.4)	9.3 (0.4)	9.5 (0.4)	0.89	0.40	0.74
T60	8.9 (0.3)	9.2 (0.3)	9.1 (0.3)	9.3 (0.3)	0.6	0.32	0.87
T90	8.0 (0.3)	7.9 (0.3)	8.2 (0.3)	7.8 (0.3)	0.42	0.86	0.37
T120	7.2 (0.3)	7.4 (0.2)	7.3 (0.2)	7.2 (0.2)	0.72	0.34	0.97
Glucose AUC	944 (27)	933 (43)	954 (26)	983 (22)	0.44	0.51	0.41
Insulin (ρmol/l)							
T0	318 (29)	432 (24)*	433 (31)*	282 (30)^††^	0.57	0.54	**0.001**
T15	1365 (96)	1569 (89)	1437 (100)	1823 (96)^†^	0.10	**0.004**	0.35
T30	969 (109)	1296 (110)*	1417 (114)*	1301 (99)	**0.05**	0.34	**0.05**
T60	689 (90)	935 (95)*	1116 (86)*	1086 (89)	**0.003**	0.25	0.15
T90	427 (66)	525 (69)	734 (72)*	558 (65)^†^	0.09	0.87	0.22
T120	294 (44)	374 (40)	580 (46)**	334 (38)^††^	**0.01**	0.08	**0.001**
Insulin AUC	59.4 (2.5)	69.3 (3.6)*	96.0 (3.0)**	81.6 (3.8)^†^	0.16	0.10	**0.005**

Data are Means (SE). *P-values* ≤ 0.05 are bold. *p < 0.05; **p < 0.005 vs. Con-Con ^†^*p* < 0.05 vs. ^††^*p* < 0.005 HF-Con. AUC, area under the curve. p^1^ represents p-value of two-way ANOVA with factors including diet and treatment interaction.

**TABLE 7:** Indices of glucose metabolism obtained during oral glucose tolerance test performed on day 95 in the female adult offspring.

	Maternal control diet		Maternal high-fat diet		Effect of diet	Effect of treatment	Diet vs. treatment interaction
			
Female adult offspring	Control	Fish oil	Control	Fish oil	p^1^	p^1^	p^1^
	15	17	12	12			
Matsuda index	3.4 (0.8)	2.5 (0.5)*	2.3 (0.5)**	3.0 (0.9)^†^	**0.0001**	0.36	**0.005**
Glucose (mmol/l)							
T0	5.9 (0.2)	6.1 (0.2)	6.1 (0.2)	6.4 (0.2)	0.16	0.14	0.76
T15	8.8 (0.5)	8.7 (0.6)	9.8 (0.5)	9.6 (0.5)	0.09	0.85	0.93
T30	8.6 (0.4)	9.3 (0.5)	9.2 (0.4)	9.2 (0.4)	0.57	0.46	0.45
T60	8.4 (0.3)	7.5 (0.3)	8.1 (0.3)	8.5 (0.3)	0.23	0.52	0.06
T90	7.1 (0.2)	7.3 (0.3)	7.3 (0.2)	7.0 (0.2)	0.86	0.55	0.20
T120	7.0 (0.2)	7.2 (0.3)	7.1 (0.2)	6.8 (0.3)	0.55	0.39	0.85
Glucose AUC	1023 (28)	1018.2 (30)	1023 (22)	1051 (39)	0.28	0.63	0.12
Insulin (ρmol/l)							
T0	184 (19)	267 (22)**	430 (20)**	252 (19)^††^	**0.001**	**0.03**	**0.001**
T15	1272 (49)	1354 (57)	1605 (51)**	1673 (49)	**0.001**	0.16	0.9
T30	693 (58)	617 (67)	932 (61)*	877 (58)	**0.001**	0.29	0.86
T60	474 (56)	542 (65)	716 (40)**	578 (56)	0.06	0.87	0.19
T90	221 (39)	431 (45)**	666 (59)*	449 (38)^††^	**0.001**	0.48	**0.001**
T120	167 (24)	261 (27)*	421 (25)**	271 (24)^††^	**0.001**	0.26	**0.001**
Insulin AUC	82.5 (7.3)	104.9 (6.9)*	117.9 (6.2)*	112.7 (9.5)	**0.04**	0.37	**0.01**

Data are means (SE). *P-values* ≤ 0.05 are bold. *p < 0.05; **p < 0.005 vs. Con-Con ^†^*p* < 0.05; ^††^*p* < 0.005 vs. HF-Con. AUC, area under the curve. p^1^ represents *p*-value of two-way ANOVA with factors including diet and treatment interaction.

### Secondary outcomes

This were also interactions between treatment and diet for free fatty acid concentration, and amongst female offspring, cardiac outcomes [ejection fraction and fractional shortening, and adiponectin concentration (all *p* < 0.05)]. For this reason, while the overall ANOVA statistic across all 4 groups is presented in all data tables, within the text, results are presented as pair-wise between-group comparisons, to better delineate the relative impacts of the diet from the treatment (with *p*-values representing the *post hoc* 2-way comparisons with Holm-Sidak’s correction). These were (1) HF-Con vs. Con-Con, to identify the effect of maternal HFD in pregnancy and lactation, (2) HF-FO vs. HF-Con, to identify the effect of maternal FO supplementation in the context of the HFD diet, and (3) Con-FO vs. Con-Con to identify the effect of maternal FO supplementation in the context of a control diet. Offspring weight gained and food consumption after weaning is presented in Figures 2B,C.

#### Effect of maternal high fat diet (comparison of HF-Con and Con-Con groups)

##### Maternal effects

At GD20, within the culled subset of the pregnant rats, there were no differences between the HF-Con and Con-Con groups in any biomarkers, including body weight, relative liver, and fat pad weight, or metabolic measures including HOMA-IR and triglycerides (Table 4).

All remaining mothers were assessed at the PN21 cull. The relative liver weight was smaller in the HF-Con group (4.1 vs. 6.4% of body weight, *p* = 0.04). In addition, there was a trend toward greater gonadal and retroperitoneal fat mass in the HF-Con group (*p* = 0.07 and *p* = 0.06, respectively).

##### Offspring effects

Among fetuses at GD20 and newborn pups on Day 1, there were no between-group differences in any outcome (Tables 3, 4). On PN2, female pups of the HF-Con group had greater relative liver weights than the Con-Con group (5.3 vs. 3.8% of body weight, respectively; *p* = 0.0002). There were no differences in blood glucose in either sex.

At PN21, in comparison to the Con-Con group, male weanlings of the HF-Con group were heavier (65.5 vs. 56.2 g, *p* = 0.001), and had greater relative gonadal and retroperitoneal fat weights (0.4 vs. 0.2% of body weight, *p* = 0.0001 and 0.6 vs. 0.4 g % of body weight, *p* = 0.0001, respectively). Similarly, female weanlings of the HF-Con group were heavier (63.1 vs. 54.4 g, *p* = 0.0001) and had greater relative gonadal fat and retroperitoneal fat weights (0.5 vs. 0.3 g % of body weight, *p* = 0.0001 and 0.4 vs. 0.3 g % of body weight, *p* = 0.0001, respectively). There were no differences in relative liver weights between groups (Table 5).

When the adult phenotype was assessed, there were no differences in body composition (percentage body fat) in either sex. Systolic blood pressure for male offspring was higher in the HF-Con than in the Con-Con group (129.4 vs. 117.2 mmHg, *p* = 0.035). From weaning to PN100, the male offspring of the HF-Con group had greater absolute energy intake (83.5 vs. 76.7 kcal/day, *p* = 0.0003) and cumulative food intake (1860 vs. 1710 g, *p* = 0.0001) (Table 8), but there was no difference in energy intake relative to body size (i.e., kcal/g of body weight/day). No differences in any cardiac parameters in either sex reached significance (Table 9). However, there were trends to lower stroke volume within male offspring, and lower ejection fraction and greater systolic diameter amongst female offspring (*p* < 0.07).

**TABLE 8:** Adult metabolic and body composition assessments.

			Maternal control diet		Maternal high-fat diet		Effect of diet	Effect of treatment	Diet vs. treatment interaction
					
	Time		Control	Fish oil	Control	Fish oil	p^1^	p^1^	p^1^
**Male offspring**			15	17	12	12			
Post fast-food consumption	70 d	Food consumed (g)	33.4 (0.8)	38.1 (0.6)	36.9 (0.6)	37.1 (0.7)	0.07	**0.001**	**0.002**
Food intake		Energy intake (kcal/day)	76.7 (0.8)	83.6 (1.0)	83.5 (1.1)**	83.6 (0.7)	**0.0001**	**0.006**	**0.002**
		Energy intake per body weight (kcal/g/day)	0.3 (0.01)	0.3 (0.01)	0.3 (0.01)	0.3 (0.01)	0.23	0.44	0.44
		Cumulative food intake (g)	1710 (20)	1863 (23)	1860 (25)**	1862 (17)	**0.0001**	**0.006**	**0.002**
Auxology	80 d	NA length (mm)	242.9 (4.3)	254.4 (3.5)	254.0 (3.0)	255.0 (2.3)	0.72	0.37	0.23
		NT length (mm)	430.1 (6.1)	446.7 (3.2)*	443.8 (3.2)	444.8 (3.5)	0.88	0.34	0.16
		Total body fat (%)	34.4 (1.1)	34.9 (1.1)	36.3 (1.5)	34.8 (1.2)	0.91	0.84	0.78
		Fat mass to lean mass ratio	0.53 (0.03)	0.54 (0.03)	0.58 (0.04)	0.54 (0.03)	0.97	0.68	0.80
Systolic blood pressure	90 d		117.2 (2.5)	116.7 (3.1)	129.4 (2.7)*	126.3 (3.4)	**0.001**	0.55	0.67
**Female offspring**									
Post fast-food consumption	70 d	Food consumed (g)	25.9 (1.8)	26.8 (1.0)	23.8 (1.1)	25.1 (0.3)	0.13	0.38	0.86
Food intake		Energy intake (kcal/day)	56.7 (0.6)	57.1 (1.2)	58.2 (0.9)	59.3 (0.6)	0.11	0.52	0.95
		Energy intake per body weight (kcal/g/day)	0.3 (0.01)	0.3 (0.01)	0.3 (0.01)	0.3 (0.01)	0.13	0.55	0.18
		Cumulative food intake (g)	1263 (14)	1273 (26)	1297 (21)	1323 (13)	0.11	0.53	0.94
Auxology	80 d	NA length (mm)	220.6 (5.1)	223.3 (3.1)	218.8 (2.9)	220.8 (3.6)	0.59	0.56	0.94
		NT length (mm)	385.9 (2.9)	394.2 (4.4)	387.1 (4.9)	389.4 (4.2)	0.67	0.21	0.47
		Total body fat (%)	39.6 (1.9)	38.0 (1.2)	36.6 (2.2)	38.3 (0.8)	0.40	0.95	0.30
		Fat mass to lean mass ratio	0.67 (0.05)	0.62 (0.03)	0.59 (0.06)	0.62 (0.02)	0.38	0.81	0.34

Blood pressure was measured only in the male offspring (day 90). Data are Means (SE). *P-values* ≤ 0.05 are bold. *p < 0.05; **p < 0.005 vs. Con-Con. AUC, area under the curve. p^1^ represents *p*-value of two-way ANOVA with factors including diet and treatment interaction.

**TABLE 9:** Heart mass and 2D Echocardiography data of the subgroup of male and female adult offspring according to the maternal diet (control or high-fat diet) and the gel treatment during pregnancy [fish oil or control (no oil gel)].

	Maternal control diet		Maternal high-fat diet		Effect of diet	Effect of treatment	Diet vs. treatment interaction
			
	Control	Fish oil	Control	Fish oil	p^1^	p^1^	p^1^
*n*	8	8	7	6			
**Male adult offspring**							
Heart mass (% body weight)	0.29 (0.01)	0.31 (0.01)	0.29 (0.01)	0.31 (0.01)	0.51	0.10	0.72
2D Echocardiography							
Heart rate (BPM)	362.9 (27.4)	353.1 (11.3)	341.4 (15.5)	343.3 (10.9)	0.37	0.82	0.73
LV systolic diameter (mm/kg BW)^¥^	6.9 (1.2)	8.4 (0.6)	7.6 (0.9)	7.9 (2.7)	0.88	0.23	0.42
LV diastolic diameter (mm/kg BW)^¥^	13.9 (0.9)	14.0 (0.7)	13.3 (1.1)	13.8 (0.5)	0.65	0.71	0.78
Stroke volume (μl)	162.5 (24.7)	175.7 (15.4)	134.6 (13.4)^#^	178.7 (10.2)^‡^	0.49	**0.05**	0.39
Ejection fraction (%)	80.6 (5.0)	68.6 (3.1)*	73.4 (3.1)	72.0 (2.4)	0.58	0.06	0.14
Fractional shortening (%)	51.4 (4.9)	39.9 (2.8)	43.6 (2.6)	42.9 (2.2)	0.42	0.06	0.09
Cardiac output (ml/min)	57.8 (8.2)	61.3 (5.2)	45.4 (4.2)	61.4 (3.8)^‡^	0.30	**0.05**	0.30
**Female adult offspring**							
Heart mass (% body weight)	0.35 (0.01)	0.35 (0.02)	0.36 (0.01)	0.34 (0.01)	0.60	**0.05**	0.51
2D Echocardiography							
Heart rate (BPM)	360.6 (10.8)	366.9 (7.1)	352.6 (8.0)	386.0 (9.1)	0.59	**0.05**	0.17
Systolic diameter (mm/g BW)^¥^	10.2 (0.8)	13.7 (1.2)*	12.1 (1.3)^#^	9.7 (1.3)	0.35	0.63	**0.01**
Diastolic diameter (mm/g BW)^¥^	20.2 (1.3)	22.1 (1.5)	20.5 (1.8)	18.6 (0.7)	0.29	0.99	0.21
Stroke volume (μl)	103.9 (12.5)	140.0 (26.1)	110.4 (17.8)	98.1 (9.5)	0.38	0.55	0.23
Ejection fraction (%)	80.4 (2.3)	65.6 (4.3)*	70.9 (3.9)^#^	78.3 (4.0)	0.67	0.32	**0.007**
Fractional shortening (%)	49.8 (2.2)	37.9 (3.9)*	41.7 (3.4)	49.1 (4.7)	0.68	0.54	**0.01**
Cardiac output (ml/min)	37.3 (4.4)	51.0 (9.4)^#^	38.8 (6.3)	37.8 (3.8)	0.41	0.37	0.30

Heart mass was measured at postnatal day 100, while echocardiogram was performed on postnatal day 90. Data are Means (SE). BPM, beats per min; BW, body weight; LV, left ventricular. ^¥^Systolic and diastolic diameters were divided by body weight to obtain a ratio for analysis, so that the unit is mm/kg of body weight. *P-values* ≤ 0.05 are bold. *p < 0.05 vs. Con-Con, ^#^*p* < 0.07 vs. Con-Con, ^‡^*p* < 0.07 vs. HF-Con. p^1^ represents *p*-value of two-way ANOVA with factors including diet and treatment interaction.

At the PN100 cull, male and female offspring in the HF-Con group had increased fasting insulin concentrations as compared to the Con-Con group (335 vs. 248 ρmol/l, *p* = 0.04 and 476 vs. 137 ρmol/l, *p* = 0.005, respectively) (Tables 10, 11). However, there were no differences between groups in body weight, relative organ weights (liver, retroperitoneal fat), or plasma adipokine concentrations (leptin, adiponectin), lipid profile, or liver enzymes.

**TABLE 10:** Characteristics and metabolic parameters of male adult offspring culled on postnatal day 100 according to the maternal diet (control or high-fat diet) and the gel treatment during pregnancy [fish oil or control (no oil gel)].

	Maternal control diet		Maternal high-fat diet		Effect of diet	Effect of treatment	Diet vs. treatment interaction
			
	Control	Fish oil	Control	Fish oil	p^1^	p^1^	p^1^
*n*	15	17	12	12			
Weight (g)	457.5 (9.5)	514.2 (8.3)**	488.2 (11.6)	500.6 (8.7)	0.51	**0.001**	**0.03**
Liver weight (% body weight)	2.6 (0.1)	2.7 (0.1)	2.6 (0.1)	2.7 (0.1)	0.67	0.18	0.52
Retroperitoneal fat (% body weight)	1.6 (0.2)	1.7 (0.1)	1.9 (0.1)	1.9 (0.2)	0.09	0.24	0.97
**Glucose homeostasis**							
Fasting glucose (mmol/l)	5.8 (0.2)	6.1 (0.2)	6.1 (0.2)	6.4 (0.3)	0.19	0.12	0.85
Fasting insulin (pmol/l)	248 (44)	288 (64)	335 (76)*	320 (52)	**0.04**	0.31	0.91
HOMA-IR	3.6 (0.7)	4.7 (1.3)	5.2 (1.2)	5.1 (0.9)	**0.01**	0.38	0.89
**Biochemical analysis**							
ALP (U/l)	98.7 (4.5)	99.1 (6.8)	99.3 (7.1)	103.5 (6.6)	0.83	0.43	0.31
ALT (U/l)	46.6 (2.0)	51.0 (2.6)	49.0 (2.7)	51.5 (2.2)	0.34	0.07	0.99
AST (U/l)	130.9 (7.2)	153.5 (11.8)	142.9 (7.6)	136.7 (8.5)	0.95	0.26	0.27
Free fatty acids (mmol/l)	0.55 (0.02)	0.64 (0.03)	0.66 (0.04)	0.60 (0.03)	0.25	0.89	**0.01**
Total cholesterol (mmol/l)	1.7 (0.1)	1.7 (0.1)	1.9 (0.1)	2.1 (0.1)	**0.01**	0.33	0.25
HDL-C (mmol/l)	1.2 (0.1)	1.2 (0.1)	1.2 (0.1)	1.4 (0.1)	0.10	0.34	0.41
LDL-C (mmol/l)	0.3 (0.03)	0.3 (0.02)	0.3 (0.13)	0.4 (0.11)	**0.02**	0.81	**0.01**
Triglycerides (mmol/l)	0.7 (0.9)	0.6 (1.3)	0.6 (0.8)	0.7 (1.0)	0.99	0.06	0.58
**Other**							
Leptin (ng/ml)	4.0 (0.4)	5.3 (0.6)	4.5 (0.4)	4.8 (0.5)	0.98	0.11	0.30
Adiponectin (ng/ml)	6392 (444)	7539 (519)	6561 (488)	6182 (596)	0.19	0.17	0.38

Data are means (SE). *p* values ≤ 0.05 are bold. *p < 0.05; **p < 0.005 vs. Con-Con. p^1^ represents *p-value* of two-way ANOVA with factors including diet and treatment interaction.

**TABLE 11:** Characteristics and metabolic parameters of female adult offspring culled on postnatal day 100 according to the maternal diet (control or high-fat diet) and the gel treatment during pregnancy [fish oil or control (no oil gel)].

	Maternal control diet		Maternal high-fat diet		Effect of diet	Effect of treatment	Diet vs. treatment interaction
			
	Control	Fish oil	Control	Fish oil	p^1^	p^1^	p^1^
*n*	15	15	12	12			
Weight (g)	262.7 (5.4)	282 (6.5)	268.7 (5.8)	288.8 (11.4)	0.18	**0.03**	0.72
Liver weight (% body weight)	2.6 (0.1)	2.7 (0.1)	2.7 (0.1)	2.5 (0.1)	0.19	0.49	**0.04**
Retroperitoneal fat (% body weight)	1.3 (0.1)	1.2 (0.1)	1.2 (0.1)	1.4 (0.1)	0.68	0.40	0.08
**Glucose homeostasis**							
Fasting glucose (mmol/l)	5.3 (0.1)	5.9 (0.2)	5.2 (0.2)	5.6 (0.2)	0.28	**0.03**	0.41
Fasting insulin (pmol/l)	137 (23)	241 (54)	476 (116)**	342 (69)	**0.001**	0.43	0.15
HOMA-IR	1.9 (0.3)	3.5 (0.8)	6.0 (1.3)*	4.9 (1.1)	**0.001**	0.76	0.24
**Biochemical analyses**							
ALP (U/l)	52.3 (4.8)	68.5 (3.8)*	59.0 (3.6)	61.8 (3.9)	0.54	0.06	0.33
ALT (U/l)	43.9 (3.1)	52.4 (4.8)	41.9 (3.2)	51.2 (4.2)	0.51	0.09	0.64
AST (U/l)	129.4 (14.9)	182.8 (29.0)	129.4 (6.2)	160.1 (17.6)	0.81	**0.04**	0.81
Free fatty acids (mmol/l)	0.60 (0.03)	0.74 (0.05)	0.69 (0.03)	0.64 (0.03)	0.82	0.24	**0.01**
Total cholesterol (mmol/l)	2.1 (0.1)	2.0 (0.1)	2.1 (0.1)	2.2 (0.1)	0.39	0.93	0.47
HDL-C (mmol/l)	1.7 (0.1)	1.5 (0.1)	1.7 (0.1)	1.7 (0.1)	0.48	0.77	0.45
LDL-C (mmol/l)	0.3 (0.02)	0.3 (0.02)	0.3 (0.02)	0.2 (0.02)	0.81	0.95	0.71
Triglycerides (mmol/l)	0.70 (0.04)	0.76 (0.09)	0.71 (0.08)	0.77 (0.10)	0.88	0.32	0.42
**Other**							
Leptin (ng/ml)	2.7 (0.3)	3.4 (0.2)	2.5 (0.1)	3.2 (0.6)	0.40	**0.01**	0.96
Adiponectin (ng/ml)	7306 (658)	6695 (401)	6070 (475)	8042 (482)	0.98	0.21	**0.05**

Data are means (SE). *p* values ≤ 0.05 are bold. *p < 0.05; **p < 0.005 vs. Con-Con. p^1^ represents *p-value* of two-way ANOVA with factors including diet and treatment interaction.

#### Effect of fish oil supplementation in pregnant rats fed the high fat diet (HF-FO vs. HF-Con)

##### Maternal effects

At GD20, within the culled subset of the pregnant rats, there were no differences between the HF-FO and HF-Con groups in any outcomes, including body weight, relative liver, and fat pad weight, or metabolic measures including HOMA-IR and triglycerides (Table 3). Further, when the remaining animals were assessed at weaning (PN21), there were also no differences between groups (Table 5).

##### Offspring effects

Among the fetuses and neonates, there were no differences in any outcomes (Tables 3, 4). On PN2, the female pups of the HF-FO group had lower relative liver weights (3.7 vs. 5.3% of body weight, *p* = 0.0001) than those of the HF-Con group. Among the weanlings (PN21), there were no differences in any biomarkers including weight, relative liver, and fat pad weights or metabolic measures (glucose, insulin, or HOMA-IR) (Table 5).

There were no between-group differences in systolic blood pressure in male offspring, body composition, post-fast food consumption, or in intake of energy or food in either sex (PN21-PN100) (Table 8) or cardiac parameters (Table 9). However, there were trends to greater stroke volume and cardiac output amongst male offspring in the HF-FO group (*p* < 0.07). At PN100, there were no between-group differences in any biomarkers (Tables 10, 11).

#### Effect of fish oil supplementation in pregnant rats fed the control diet (Con-FO vs. Con-Con)

##### Maternal effects

At GD20, within the culled subset, mothers of the Con-FO group had increased insulin concentrations (392 vs. 257 ρmol/l, *p* = 0.04) and greater HOMA-IR (6.3 vs. 4.0, *p* = 0.03) than the Con-Con group (Table 4). There were no between-group differences in other biomarkers. At PN21, there were no differences in any outcomes measured (Table 5).

##### Offspring effects

There were no between-group differences in the outcomes in the fetuses culled on GD20. However, newborn male pups of the Con-FO group were heavier (6.6 vs. 6.3 g, *p* = 0.03) than those in the Con-Con group (Tables 3, 4).

At PN21, there were no differences in the weight, relative liver and fat pad weights or metabolic biomarkers between the Con-Con and Con-FO groups in either sex (Table 5).

In adulthood, there were no differences in body composition (body fat percentage) in either sex. The male offspring of the Con-FO group had longer nose-tail length (446.7 vs. 430.1 mm, *p* = 0.03) than the Con-Con group, but no difference in nose-anal length. There were no differences in the systolic blood pressure in the males and food intake in either sex (Table 8). However, FO supplementation to dams fed a control diet led to reduced ejection fraction in both the male (68.6 vs. 80.6%, *p* = 0.04) and female (65.6 vs. 80.4%, *p* = 0.04) adult offspring. Further, among female offspring it also led to greater systolic diameter (0.014 vs. 0.010 mm, *p* = 0.05) and reduced fractional shortening (37.9 vs. 49.8%, *p* = 0.04) (Table 9).

At PN100, males in the Con-FO group were heavier (514.2 vs. 457.5 g, *p* = 0.002) than the Con-Con group (Table 10). However, there were no differences in metabolic biomarker measures (glucose, insulin, HOMA-IR, leptin, and adiponectin) in males or females. Females in the Con-FO group had higher ALP concentrations (68.5 vs. 52.3 U/L, *p* = 0.03) than the Con-Con group but no difference in liver enzymes was observed in males (Table 11).

## Discussion

In this study, maternal consumption of a HFD during pregnancy and lactation resulted in reduced insulin sensitivity in the adult offspring and, in male offspring, greater food intake and increased systolic blood pressure. Importantly, in dams fed a HFD diet during pregnancy, treatment with a human-relevant dose of FO prevented the development of insulin resistance induced by the maternal diet in both male and female offspring.

Surprisingly, there were negative consequences of maternal FO supplementation when the dam consumed a control diet, including insulin resistance during pregnancy, an offspring phenotype of greater body weight and insulin resistance, and altered cardiac function (reduced ejection fraction). While this effect of maternal FO has not previously been reported, this observation is in general agreement with previous observations where the effects of an intervention (e.g., neonatal leptin administration) have been reported to be directionally dependent upon prior maternal nutritional status (40).

While the maternal HFD induced insulin resistance in the adult offspring, this effect was prevented by concomitant treatment of dams with dietary FO. This is consistent with previous studies of maternal HFD in the rat, where a high dose of FO (17), or intrinsic production of n-3 PUFAs (FAT-1 mouse) (41), prevented the development of insulin resistance in offspring. However, the present study is the first to show such benefits with a smaller, human-relevant dose of dietary FO, with a low peroxide value (determined independently of the manufacturer).

It is interesting that the FO treatment during pregnancy of dams consuming a high fat diet led to improvements in the Matsuda index in the adult offspring, but not to HOMA-IR. As the Matsuda index is based on the response to a glucose load, it is influenced more strongly by peripheral insulin sensitivity than HOMA-IR (42). HOMA-IR is derived from fasting glucose and insulin concentration, so largely reflects hepatic insulin sensitivity (42). This raises the possibility that the maternal FO treatment has had long-term effects on the metabolic function of skeletal muscle.

We hypothesized that the mechanism, by which maternal n-3 PUFA supplementation would prevent the offspring of mothers consuming a HFD from developing an adverse metabolic phenotype, would be through their insulin sensitizing (28) and triglyceride lowering effects (29). Alternatively, n-3 PUFAs could have crossed the placenta (43) and had direct effects on the fetus. Interestingly, we did not demonstrate improved insulin sensitivity or lowered triglycerides in the pregnant rats. This raises the possibility that the beneficial effect described was not mediated by improved maternal metabolism and was instead a direct effect of n-3 PUFAs transferred to the fetus. However, insulin resistance was estimated using HOMA-IR, which has limitations (36), and it is possible that true differences could have been detected if a glucose tolerance test or a direct measure of insulin sensitivity (e.g., hyperinsulinemic euglycemic clamp) had been used. Nevertheless, it is clear that the offspring phenotype was improved by the maternal FO supplement.

We did not demonstrate an adverse effect of maternal HFD on cardiac morphology or function in the adult offspring. An effect may have been expected, as hyperinsulinaemia in offspring exposed to a maternal HFD is thought to induce adverse changes in cardiac morphology (26). However, though differences did not reach significance, there were trends to greater systolic diameter, lower ejection fraction, and lower stroke volume in the HFD offspring. Interestingly, there was also a trend to improved stroke volume and cardiac output in the offspring of HFD fed dams treated with FO. This would be consistent with the FO treatment partially ameliorating the effects of the maternal HFD on offspring cardiovascular function. A study with a larger sample size, would be required to better understand the relationship between maternal HFD and FO consumption on cardiovascular function in offspring.

This is the first study to report a negative impact of supplementation with FO during normal pregnancy on mothers and their offspring. Amongst mothers consuming a control diet, supplementation with FO led to reduced insulin sensitivity during pregnancy. Consequently, adult offspring had an adverse metabolic phenotype with hyperinsulinaemia and reduced insulin sensitivity. Further, there was an adverse effect on cardiac morphology and function in the adult offspring, which may have been mediated by hyperinsulinaemia (26). To our knowledge, such adverse programming effects of FO in normal pregnancy have not been previously reported.

The mechanism for the adverse effect of FO supplementation to dams consuming a control diet is not clear. However, it is plausible that the relatively high n-6 PUFA content of the control diet could have contributed, leading to greater oxidative stress (44) in the dams and/or fetuses and limiting beneficial effects of n-3 PUFAs by competing for transport and metabolism to biologically active mediators (45). Future studies using a control feed rich in monounsaturated fatty acids instead of PUFAs could help to understand whether n-6 PUFAs in feed contributed to the harmful effects of the fish oil in the context of a maternal control diet.

Our data suggests that the effect of the maternal FO supplement on the offspring’s metabolic phenotype depends on the metabolic context of the mother. When the mother was metabolically compromised by consuming a HFD, FO had beneficial effects on the adult offspring, but in the context of a control diet, modeling normal pregnancy, the effects of the FO on phenotype were opposite. These diametrically opposed effects may have important implications for supplementation in human pregnancy. Studies of FO supplementation in human pregnancy have not revealed clear effects of the maternal supplement on offspring body composition or metabolism (46). However, such studies were conducted in women predominantly of normal BMI (46). The maternal HFD was used to model obese pregnancy as it has similar direct metabolic effects on the mother (20), and similar effects on offspring metabolism (21, 22). This suggests that FO treatment of women who are overweight or obese during pregnancy could have discrepant and more beneficial effects than supplementation of normal weight women. The greatest strength of this study was in the balanced factorial design that allowed us to determine and compare the effects of the maternal FO intervention in the context of a HFD with those in the control diet. In addition, the offspring were assessed in adulthood, so that potential differences in metabolism that emerge with age could be identified, and the study was adequately powered to allow sex-specific analyses. Importantly, a human-relevant dose of FO was used, indicating that the large doses previously reported (17) are not necessary to induce long term metabolic benefits. Lastly, the oil was independently verified for n-3 PUFA content and oxidative state and delivered using small, emulsified gels, which act as an enriching vehicle that enables rapid delivery of a controlled dose (33).

This study also had limitations. Firstly, in part, due to higher-than-expected rates of non-pregnancy, relatively few animals were culled for assessment during pregnancy, thus we may have had insufficient power to detect between-group differences at that time point. Secondly, we did not assess insulin sensitivity using the hyperinsulinemic euglycemic clamp, which is the gold standard method and would have enabled us to better understand the relative contributions of hepatic and peripheral insulin sensitivity to the metabolic phenotype. In addition, we were not able to assess blood pressure in female offspring. Lastly, in this study the intervention was started immediately after conception, which would be difficult to translate to human supplementation, where pregnancy is often not recognized until many weeks after conception.

In conclusion, this is the first study to show that maternal FO supplementation at a human-relevant dose, in the context of a HFD, has beneficial effects on the offspring metabolic phenotype, suggesting that similar benefits might be achieved in the context of maternal obesity in women. In contrast, FO supplementation in normal dams (i.e., those consuming a control diet) led to both maternal insulin resistance and an adverse metabolic phenotype in adult offspring, as well as adverse changes in cardiac morphology and function. These divergent findings based on maternal nutritional status demonstrate the importance of the metabolic context when considering use of a treatment in pregnancy that affects metabolism. We speculate that FO supplementation in pregnancy may have greater benefits in women with overweight or obesity than in those within the healthy weight range.

## Data availability statement

The raw data supporting the conclusions of this article will be made available by the authors, without undue reservation.

## Ethics statement

The animal study was reviewed and approved by Animal Ethics Committee, University of Auckland.

## Author contributions

BA, MV, and WC conceived the study and won the funding for the study. VS, BA, MV, CR, EF, and AP carried out the study. MG analyzed the oil fatty acid composition. AP and CB analyzed the echocardiograms. VS analyzed the data. VS, BA, and MV interpreted the data. VS, BA, and MV drafted the manuscript. All authors contributed to the final manuscript.
